# Reactive Solubilization of Heterometallic Clusters by Treatment of (TrBi_3_)^2−^ Anions (Tr=Ga, In, Tl) with [Mn{N(SiMe_3_)_2_}_2_]

**DOI:** 10.1002/anie.202210683

**Published:** 2022-09-15

**Authors:** Julia Rienmüller, Andreas Schmidt, Nathan J. Yutronkie, Rodolphe Clérac, C. Gunnar Werncke, Florian Weigend, Stefanie Dehnen

**Affiliations:** ^1^ Fachbereich Chemie and Wissenschaftliches Zentrum für Materialwissenschaften (WZMW) Philipps-Universität Marburg Hans-Meerwein-Str. 4 53043 Marburg Germany; ^2^ Univ. Bordeaux CNRS Centre de Recherche Paul Pascal CRPP UMR 5031 33600 Pessac France

**Keywords:** Binary Zintl Anions, Bismuth, DFT Calculations, Manganese Compounds, Triel Elements

## Abstract

Lowering the charge of Zintl anions by (element‐)organic substituents allows their use as sources of (semi)metal nanostructures in common organic solvents, as realized for group 15 anions or Ge_9_^4−^ and Sn_9_^4−^. We developed a new strategy for other anions, using low‐coordinate 3d metal complexes as electrophiles. [K(crypt‐222)]^+^ salts of (TrBi_3_)^2−^ anions dissolved in situ in Et_2_O and/or THF when reacted with [Mn(hmds)_2_]. Work‐up afforded soluble [K(crypt‐222)]^+^ salts of [{(hmds)_2_Mn}_2_(TlBi_3_)]^2−^ (in **1**), [{(hmds)_2_Mn}_2_(Bi_2_)]^2−^ (in **2**), and [{(hmds)Mn}_4_(Bi_2_)_2_]^2−^ (in **3**) (crypt‐222=4,7,13,16,21,24‐hexaoxa‐1,10‐diazabicyclo[8.8.8]hexacosane; Tr=Ga, In, Tl; hmds=N(SiMe_3_)_2_), representing rare cases of Zintl clusters with open‐shell metal atoms. **1** comprises the first coordination compound of the (TlBi_3_)^2−^ anion, **2** features a diamond‐shaped {Pn_2_M_2_} unit, and **3** is a mixed‐valent Mn^I^/Mn^II^ compound. The uncommon electronic structures in **1**–**3** and magnetic coupling were studied by comprehensive DFT calculations.

## Graphical abstract

The otherwise insoluble binary Zintl anions (TrBi_3_)^2−^ (Tr=Ga, In, Tl) can be solubilized in THF on treatment with the low‐coordinate manganese complex [Mn(hmds)_2_] (hmds=N(SiMe_3_)_2_), yielding new heterometallic Zintl clusters [{(hmds)_2_Mn}_2_(TlBi_3_)]^2−^, [{(hmds)_2_Mn}_2_(Bi_2_)]^2−^ (see figure), and [{(hmds)Mn}_4_(Bi_2_)_2_]^2−^. The electronic properties of the Bi_2_^2−^ anion‐containing coordination compounds are disclosed.

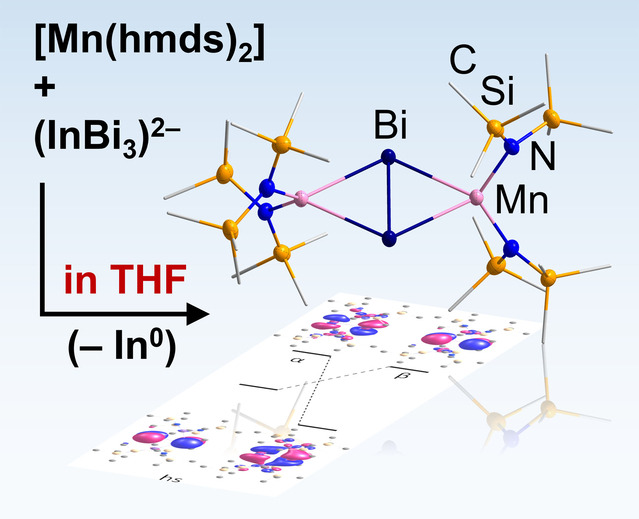

## Introduction

In recent years, Zintl anions have been proven excellent starting materials of heterometallic and intermetalloid clusters, in which p‐block (semi)metal atoms are combined with d‐block or f‐block metal atoms.[^1^, ^2^, ^3^, ^4^, ^5^] However, owing to their intrinsic anionic charges and sensitivities, the access and further treatment of such species have been widely restricted to very few, highly basic solvents—ethane‐1,2‐diamine (en), liquid ammonia, or N,N‐dimethylformamide (dmf). The attachment of elementorganic or organic groups served to lower the negative charge, and therefore allowed for solubility in common solvent like THF or CH_3_CN. This has been extensively applied to Zintl anions of group 15 elements,^[6]^ and later on, in 2007, also for Zintl anions of group 14 elements with their intrinsically higher charge per atom.[^3^, ^7^] Some years later, in 2012, full compensation of the charge even allowed for water solubility and compatibility, which opened up new options for subsequent reactions.^[8]^ However, so far, such modifications have only been feasible for the homoatomic Zintl anions Ge_9_^4−^ and Sn_9_^4−^.

Another way of lowering the charge, which was successfully applied to Ge_9_^4−^ and Sn_9_^4−^,[^9^, ^10^, ^11^] but also to Si_4_^4−^, Ge_4_^4−^, (Si_4‐*x*_Ge_*x*_)^4−^, and Sn_4_^4−^, with even higher charges per atom, is the use of Zintl anions as ligands of transition metal complexes; in these cases, the nine‐atom or four‐atom cages act as Lewis bases that replace one or more of the original ligands of the transition metal complex. Products of such reactions have been combinations of said Zintl anions with group 6 metal carbonyl complex fragments {M(CO)_3_} (M=Cr, Mo, W)^[12]^ and d^7^ or d^10^ metal complex fragments, such as {Ir(cod)}, {CuL} (L=Mes, NHC^Dipp^, P^i^Pr_3_, PCy_3_), {PdPPh_3_}, {ZnL} (L=Ph, Mes, ^i^Pr) or {CdL} (L=Ph, Sn^n^Bu_3_) units,[^9^, ^10^, ^11^, ^13^, ^14^, ^15^] or with “naked” cations of groups 11 and 12.[^13^, ^15^, ^16^, ^17^] Some of the latter have also been applied to binary Zintl anions, which have been reported to form anions like [(PhZn)_2_(Sn_2_Sb_5_)]^3−^,^[18]^ [{(cod)Ru}(Tl_2_Bi_6_)]^2−^ (cod=1,5‐cyclooctadiene)^[19]^ with remaining organic ligands, as well as [Au(Sn_2_Sb_2_)_2_]^3−^ and the one‐dimensional strand {[Au(TlSn_3_)]^4−^}_*n*_ without ligands.[^17^, ^20^] All of these species actually represent bridges to the heterometallic and intermetallic clusters mentioned above because they do not differ from the original Zintl anions regarding their solubility, and thus will not be detailed here.[^1^, ^2^, ^3^, ^4^, ^5^]

In summary, binary anions with group 13/15 or group 14/15 elemental combinations could not yet be systematically transferred into well‐soluble derivatives with organic shielding. For [K(crypt‐222)]^+^ salts of (TrBi_3_)^2−^ anions (crypt‐222=4,7,13,16,21,24‐hexaoxa‐1,10‐diazabicyclo[8.8.8]hexacosane; Tr=Ga, In, Tl), one of the underlying problems is the inclination of (TrBi_3_)^2−^ to undergo redox processes, in which the formally negatively charged Tr atoms are released as elemental metals, while polybismuthide units would be integrated in usually ligand‐free cluster molecules;^[21]^ thus the choice of a suitable reactant must be done with even greater care.

In the light of the described state‐of‐the‐art, we recognize an obvious lack of highly versatile synthetic approaches in common solvents. However, such methods would provide significant benefit for multimetallic cluster syntheses in general. Inspired by this and by the corresponding experimental challenge, we developed a new way of shielding Zintl anions, which we report herein. Our synthetic strategy is based on the idea of using the two‐coordinate transition metal complex [Mn(hmds)_2_] (hmds=N(SiMe_3_)_2_) as strong, yet electrochemically relatively inert, electrophiles for attachment to Zintl anions.

## Results and Discussion

**Figure 1:**
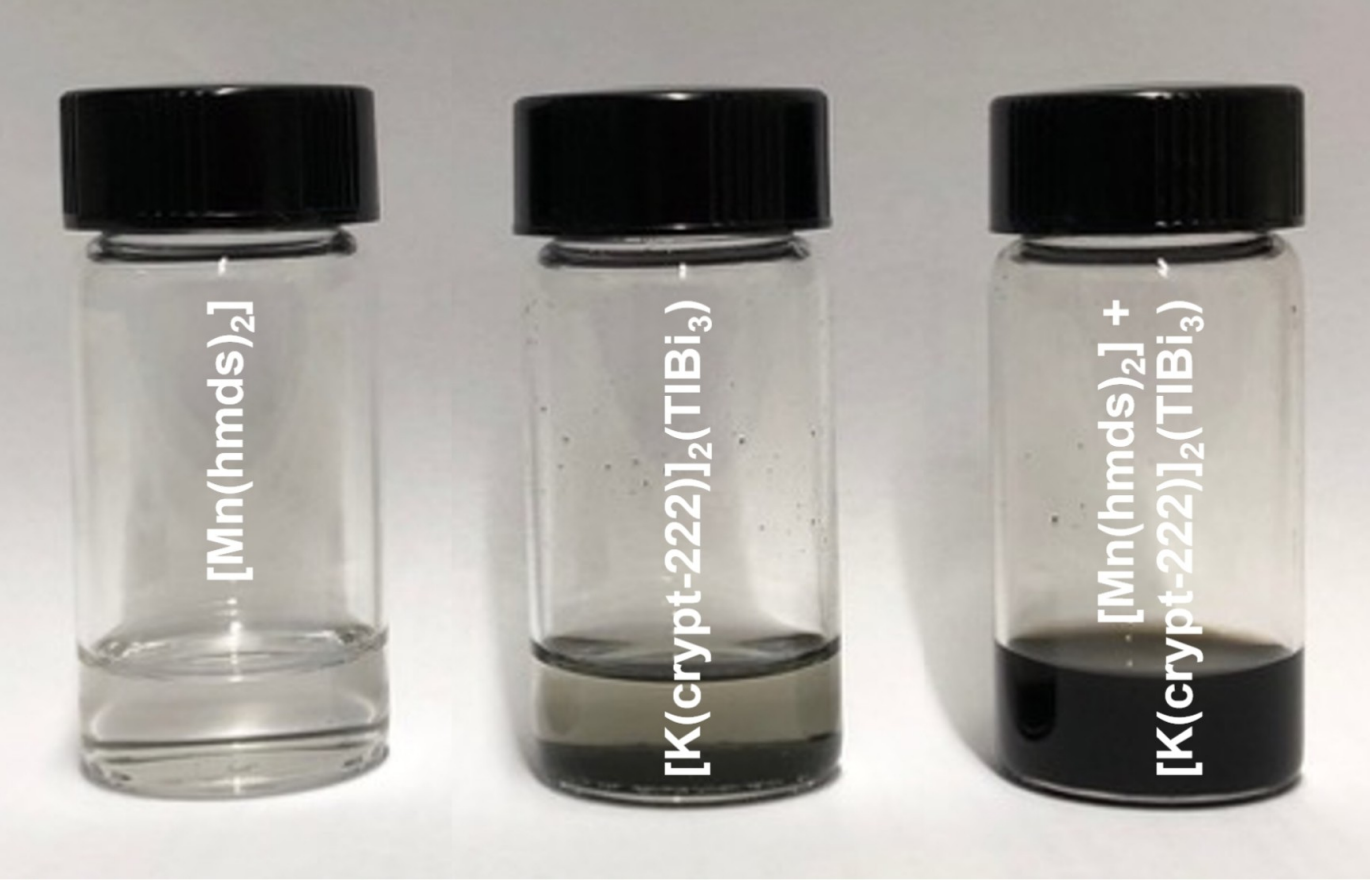
Photographs illustrating the behavior of the reactants in THF: solution of [Mn(hmds)_2_] in THF (left), suspension of [K(crypt‐222)]_2_‐(TlBi_3_) ⋅ en in THF (center) and reactive solution of [Mn(hmds)_2_]+[K(crypt‐222)]_2_(TlBi_3_) ⋅ en in THF (right).

A suspension of [K(crypt‐222)]_2_(TrBi_3_)⋅en (Tr=Ga, In, Tl) in THF or Et_2_O/THF mixtures (v:v=1 : 1) immediately turned dark brown upon addition of [Mn(hmds)_2_], indicating reactive dissolution of the Zintl salt (see Figure 1).

**Scheme 1:**
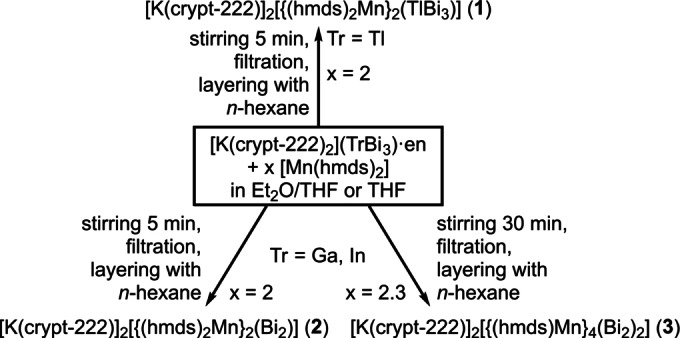
Non‐stoichiometric reaction schemes for the syntheses of **1**–**3**. Crystal solvent is not indicated here for clarity. The nature and amount of crystal solvents are given in the text. For more details see the Supporting Information.

From such solutions, we obtained the novel compounds [K(crypt‐222)]_2_[{(hmds)_2_Mn}_2_(TlBi_3_)]⋅1.5 Et_2_O (**1**⋅1.5 Et_2_O), [K(crypt‐222)]_2_[{(hmds)_2_Mn}_2_(Bi_2_)]⋅4 THF (**2**⋅4 THF), and [K(crypt‐222)]_2_[{(hmds)Mn}_4_(Bi_2_)_2_] (**3**) upon filtration and layering with *n*‐hexane, as summarized in Scheme 1.

All three compounds were investigated by means of single‐crystal X‐ray diffraction^[22]^ and by elemental analyses using micro‐X‐ray fluorescence spectroscopy (μ‐XFS), which confirmed their composition (see Supporting Information). In contrast, electrospray‐ionization mass spectrometry (ESI‐MS) was not successful on any of these compounds—including the complex [Mn(hmds)_2_] itself that was used for the reactions—in spite of many attempts and a lot of experience we have with this method. In the mass spectra, we see fragments only; this clearly indicates the high tendency of hmds‐decorated molecules towards decomposition under ESI‐MS conditions.

**Figure 2:**
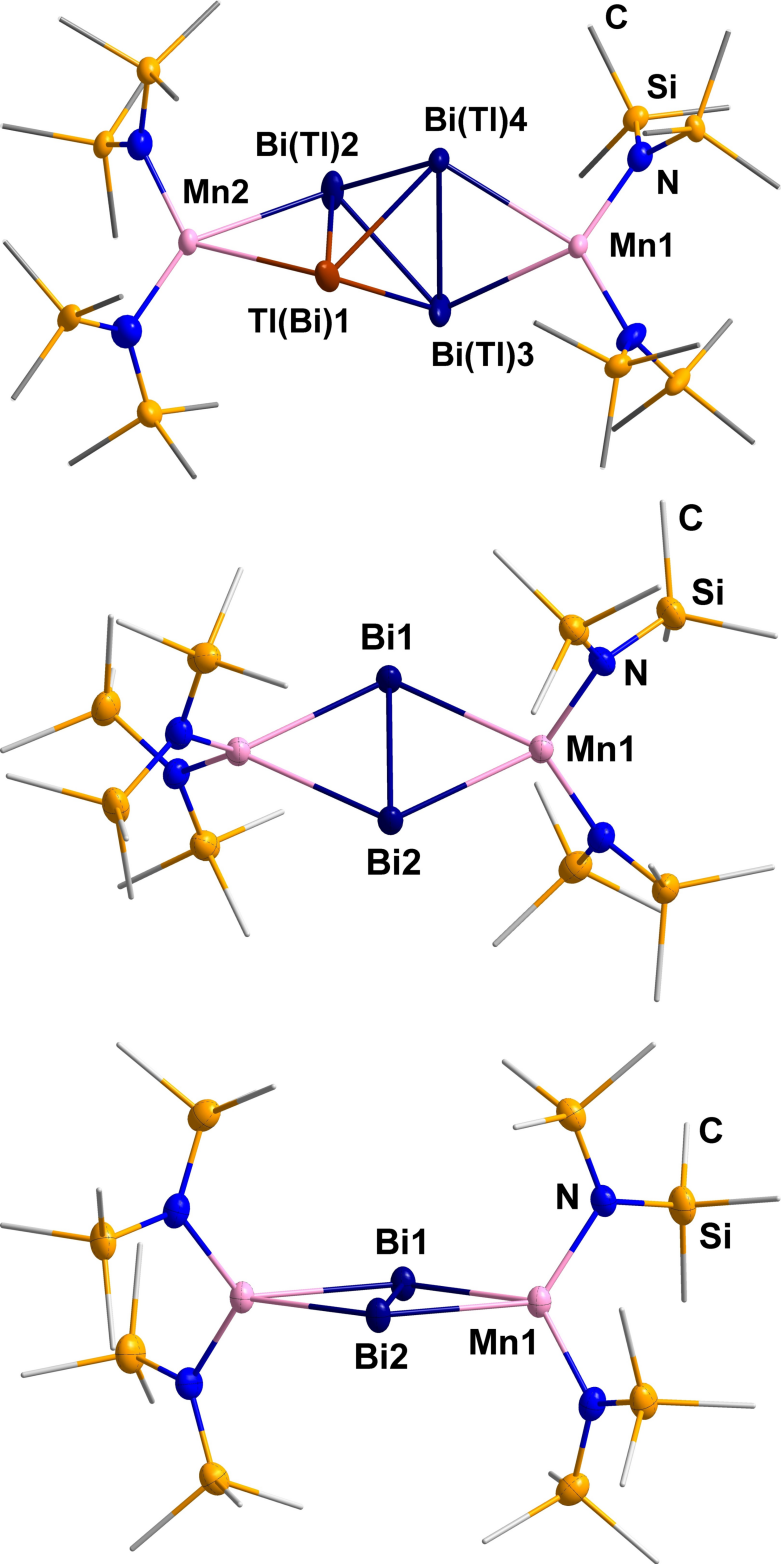
Molecular structures of the anions in compounds **1** (top) and **2** (two views: center and bottom), with Bi, Mn, N, and Si atoms shown as thermal ellipsoids with 50 % probability, C atoms are drawn as wires, H atoms are omitted for clarity. The anion in **1** shows a statistical disorder of the {TlBi_3_} unit that was modeled with two sets of split positions. As Tl and Bi atoms cannot be distinguished by standard X‐ray diffraction experiments, all involved sites were assigned a 0.125 occupancy by Tl atoms and a 0.375 occupancy by Bi atoms. For clarity, we illustrate only one of these sets here, with one of the atoms being randomly picked to represent the Tl atom (see Supporting Information for more details). Selected distances in **1** [Å]: Bi−(Tl)Bi 2.907(4)–3.311(3), Bi(Tl)−Mn 2.967(3)–3.097(3), Mn−N 2.037(3)–2.051(3). Selected distances in **2** [Å]: Bi−Bi 2.90222(18), Bi−Mn 2.9616(3)–2.9712(3), Mn−N 2.0659(19)–2.0692(18).

The molecular structures of the anion in compounds **1** and **2** are shown in Figure 2. Supplementary crystallographic figures are provided in the Supporting Information. The anion in **1** consists of a disordered (TlBi_3_)^2−^ tetrahedron which coordinates in a trans‐η^2^‐η^2^ fashion to two [Mn(hmds)_2_] units. The atomic distances within the *pseudo‐*tetrahedron are between 2.907(4) and 3.314(5) Å, with the longest distances observed at the edges that bind to the formally neutral [Mn(hmds)_2_] units. The distances are elongated on average in comparison with the free (TlBi_3_)^2−^ anion (3.04589(7)–3.0772(6) Å),^[19]^ which again reflects the involvement of the electrons from bonding orbitals in the coordination of the metal complexes. The [Mn(hmds)_2_] unit as a whole was attached to the (TlBi_3_)^2−^ anion without affecting its charge. The much better solubility of the resulting molecule is thus solely due to the four terminal SiMe_3_ groups.

While the (TlBi_3_)^2−^ anion remained intact during formation of **1**, the (GaBi_3_)^2−^ and (InBi_3_)^2−^ anions fragmented upon electrophilic attack, despite an entirely analogous reaction. Instead, both decomposed to yield elemental Ga or In, and a Bi_2_^2−^ dumbbell. Under the workup conditions indicated in Scheme 1, this Bi_2_^2−^ unit μ‐η^2^‐bridges two [Mn(hmds)_2_] units, again forming a well‐soluble molecular complex in **2**. The uncoordinated Bi_2_^2−^ anion observed in [K(crypt‐222)]_2_Bi_2_ formally possesses a double bond, with a Bi−Bi distance of 2.8377(7) Å,^[23]^ which is expanded in **2** to 2.90222(18) Å. This observation can be explained by the twofold side‐on‐coordination of the Mn^2+^ ions and a corresponding decrease of the bond order.

The described different behavior of these binary Zintl anions is in line with the observations reported in previous studies, in which the tendency to include the group 13 element in the products cluster decreases with decreasing atomic number (Tl>In>Ga). A molecular structure of the (GaBi_3_)^2−^ anion indeed has never been crystallographically determined (while the anion was detected by mass spectrometry),^[24]^ but it is supposed to be isostructural to the known (InBi_3_)^2−^ and (TlBi_3_)^2−^ anions.[^19^, ^25^] Besides this anion, two larger anions have recently been reported, (Ga_2_Bi_16_)^4−^ and [Bi@Ga_8_(Bi_2_)_6_]^3−/5−^,^[24]^ with the latter being similar to [Bi@In_8_(Bi_2_)_6_]^3−/5−^.^[26]^ Calculations revealed that (GaBi_3_)^2−^ is at the extreme limit of isolatable *pseudo*‐tetrahedral anions due to geometrical aspects, while this is less problematic for (InBi_3_)^2−^ and not a problem at all for (TlBi_3_)^2−^ due to more similar atomic sizes.^[27]^ Consequently, only few clusters have so far been reported, in which Ga or In and Bi coexist, such as [Sm@Ga_3−*x*_H_3−2*x*_Bi_10+*x*_]^3−^ (*x*=0, 1)^[28]^ or {[La@In_2_Bi_11_]_2_Bi_2_}^6−^,^[29]^ while there are several examples for the TlBi‐elemental combination. These were reported as salts of binary anions, like [K(crypt‐222)]_2_‐(TlBi_3_), [K(crypt‐222)]_3_(Tl_4_Bi_5_), and [K(crypt‐222)]_3_‐(Tl_4_Bi_3_),^[30]^ or as salts of ternary clusters like [{Ru(cod)}_4_Bi_18_]^4−^, [U@Bi_12_]^3−^, [Th@Bi_12_]^4−^, [{Ru(cod)}Tl_2_Bi_6_]^2−^, [U@Tl_2_Bi_11_]^3−^, or [Th@Tl_2_Bi_11_]^3−^, which were obtained from reactions of (TlBi_3_)^2−^ with d‐block or f‐block metal compounds [Ru(cod){H_2_CC(Me)CH_2_}_2_], [UCp^#^_3_] or [ThCp^#^_3_]Cl, for instance.[^19^, ^24^, ^31^]

We were interested to get insight into the bonding situations of the anions in compounds **1** and **2**. For this, density functional theory (DFT) calculations^[32]^ were carried out with the TPSSh functional^[33]^ and def2‐TZVP basis sets.^[34]^ In all calculations the conductor‐like screening model was employed for charge compensation.^[35]^ For exploring the binding energy (*E*_B_) between (TlBi_3_)^2−^ and two [Mn(hmds)_2_] units in the anion of **1**, we calculated the difference of the total energy of **1** and the total energies of said units. *E*_B_ amounts to −69.2 kJ mol^−1^ (thus −34.6 kJ mol^−1^ per side), both for a high‐spin state (hs; *S*=5) and for the broken‐symmetry low‐spin state (bs; *S*=0). This indicates a negligible coupling between the *S*=5/2 Mn^II^ spins. In addition, we find that the interaction between (TlBi_3_)^2−^ and Mn is highly ionic in nature. This is evident when replacing (TlBi_3_)^2−^ with isoelectronic but neutral Bi_4_ which results in an even slightly positive binding energy (+3.5 kJ mol^−1^). The Tl−Bi bonds within the *pseudo‐*tetrahedral subunit are strongly polarized. About 75 % of the Mulliken charge in the corresponding localized orbitals is assigned to Bi, and 25 % to Tl—in agreement with the higher electronegativity of Bi (1.67) as compared to Tl (1.44).^[36]^ The electronic overload at the Bi atoms is partly transferred to the attached [Mn(hmds)_2_] units, as indicated by the change in the average Mulliken charge of Bi from −0.57 in (TlBi_3_)^2−^ to −0.38 in **1**, as well as in the average Mulliken number of unpaired electrons for Mn being reduced from 5.02 for an isolated [Mn(hmds)_2_] complex to 4.89 in **1**.

**Figure 3:**
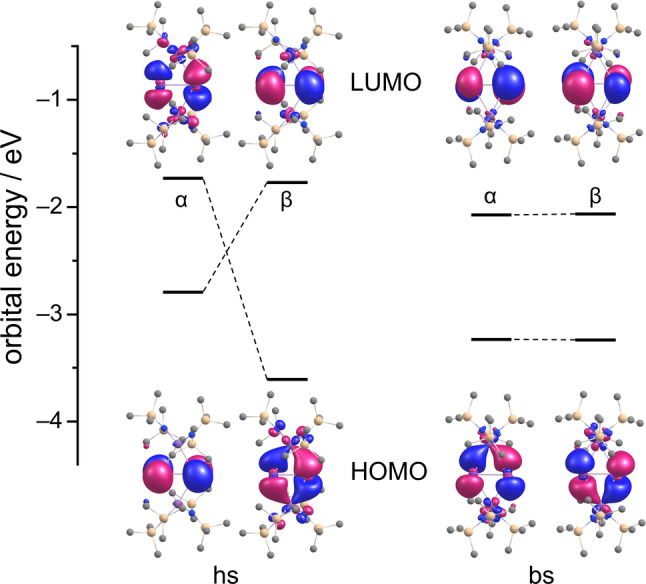
Frontier molecular spin orbitals of the anion in **2** for the high‐spin (hs) and the broken‐symmetry (bs) case. Amplitudes are drawn at ±0.035 a.u.. For hs, α refers to the majority spin, that is, for the spin of the d electrons at the Mn atoms.

The binding energy between Bi_2_^2−^ and the two [Mn(hmds)_2_] units in the anion of **2** amounts to −156.1 kJ mol^−1^ (−78 kJ mol^−1^ per side) for the high‐spin state (a second conformer with a broken‐symmetry low‐spin state is disfavored by 6.7 kJ mol^−1^, see also below). As discussed for **1**, the Bi_2_^2−^ unit and the two [Mn(hmds)_2_] moieties also undergo mainly ionic interactions, evident from the slightly positive *E*_B_ (+14.6 kJ mol^−1^) for isoelectric but neutral Te_2_. The electronic overload is again transferred to the Mn atoms, evident from the reduction of the Mulliken charge at Bi from −1 in parent Bi_2_^2−^ to −0.58 in **2**, and a reduction of the Mulliken number of unpaired electrons at the Mn atoms to 4.69. The charge transfer is also visible from the delocalization of the in‐plane parts of the π and π* orbitals of Bi_2_^2−^ to the s and d orbitals of the two Mn atoms, as shown for the π* orbitals in Figure 3. The concomitant reduction of the Bi−Bi bond order (Wiberg indices: 1.99 in Bi_2_^2−^, 1.31 in the anion of **2**) agrees well with a slightly increased Bi−Bi distance both in the calculation (by 0.034 Å) and the experiment (by 0.065 Å) relative to the bond length in Bi_2_^2−^ (calculated: 2.844 Å, experiment: 2.838 Å).^[23]^

It is noteworthy that planar, diamond‐shaped {Pn_2_M_2_} units including heavier pnictogen (Pn=As⋅⋅⋅Bi) and transition metal atoms are extremely rare,^[37]^ most likely owing to the reducing power of the Pn_2_^2−^ dianions, which prefers to end up with a pyramidal coordination environment and formally zero charge of the Pn atoms. Accordingly, the planar {Pn_2_M_2_} motif was rather observed for some rare‐earth and actinide complexes with reductively resistant metal ions and thus mainly ionic interactions ({Sm^III^_2_Pn_2_} for Pn=Bi,^[38]^ Sb,^[39]^ As,^[40]^ and {U^IV^_2_As_2_}).^[41]^ The more common motif for dinuclear transition metal compounds bearing a {μ‐η^2^‐Pn_2_} ligand is a butterfly‐shaped {Pn_2_M_2_} unit, which allows for additional M−M interactions (especially between low‐valent M atoms like in carbonyl/cyclopentadienide complexes [{Cp^Me^(CO)_2_Mo}_2_(μ‐Bi_2_)]^[42]^ and [{Cp(CO)_2_Mo}_2_(μ‐Sb_2_)]^[43]^) or for a formulation as a bridging Pn_2_^4−^ unit, as in [{(Ph_3_P)_2_Pd}(μ‐As_2_)]^[44]^ and [{(NHC)(CO)Ni}_2_(μ‐As_2_)].^[45]^ Given the scarcity of paramagnetic {Pn_2_M_2_} complexes, magnetic studies on those with open‐shell metal ions are unsurprisingly absent. For a related {Zr^III^_2_N_2_} motif bearing a bridging N_2_^2−^ unit, strong antiferromagnetic coupling was proposed,^[46]^ whereas in some paramagnetic rare‐earth {M_2_N_2_} compounds (M=Gd, Dy, Tb), the antiferromagnetic coupling is considerably weaker.^[47]^

We were therefore interested to explore how the planar {Mn_2_Bi_2_} unit would behave in this regard—and whether a (hypothetical) bent isomer would show different properties. DFT calculations indicated that beside the planar conformer, also a slightly bent conformer (dihedral angle 169.1°) can be observed for different magnetic situations. While the planar system results from a ferromagnetic coupling of the two ions, with an *S*=5 high‐spin (hs) ground state, the bent conformer accords with antiferromagnetic coupling and a corresponding *S*=0 broken symmetry (bs) low‐spin ground state. Both species are local minima on the energy hypersurface, with the experimentally observed planar conformer being very slightly preferred, by 4.2 kJ mol^−1^. This indicates that the coupling is weak. Furthermore, the energy surface exhibits a moderate curvature for this degree of freedom: forcing the broken‐symmetry state to planar shape requires 25.1 kJ mol^−1^, and forcing the high‐spin state to bent shape requires 9.3 kJ mol^−1^ only. Nevertheless, the two different coupling modes have impact on the shape of the frontier orbitals. In both cases, both HOMO and LUMO are dominated by the Bi_2_^2−^ π* orbitals, just like for the bare Bi_2_^2−^ unit. For the slightly bent broken symmetry case (right hand side of Figure 3), the HOMOs of both spin types are the in‐plane π* orbitals. One observes delocalization towards the empty Mn(d) spin minority orbitals: for the α‐spin π* to the upper Mn atom with empty α‐spin (and occupied β‐spin) d orbitals, and for the β‐spin π* to the lower Mn atom with empty β‐spin (and occupied α‐spin) d orbitals. For the high‐spin case in contrast, delocalization to empty Mn(d) orbitals is possible only for the minority spin, β. As a consequence, the in‐plane α‐spin π* orbital is significantly higher in energy than its β‐spin counterpart and also higher in energy than the α‐spin π* orbital perpendicular to the plane. Hence, for this state the HOMOs are the majority‐spin π* orbital perpendicular to the {Bi_2_Mn_2_} plane and the minority‐spin in‐plane π* orbital.

Despite many attempts on five different batches of **2** (see Supporting Information), the final experimental proof of the planar structure to exhibit a (weak) ferromagnetic coupling is still elusive, as the compounds are too sensitive as to produce reliable magnetic susceptibility (*χ*) data. We took great care while isolating, shipping, and preparing the samples for these measurements, but to date, none of the measurements delivered reproducible and physically meaningful results. The *χT* product at room temperature was found systematically lower than the expected value (8.75 cm^3^ K mol^−1^) for two isolated *S*=5/2 Mn^II^ spins (ranging from 6.4 and 7.9 cm^3^ K mol^−1^). Nevertheless, these experimental values, even if lower, strongly support the oxidation and spin states of the Mn metal ions. In all the samples, the *χT* product decreases when lowering the temperature. This indicates the presence of dominating antiferromagnetic interactions with a magnitude that varies strongly for the different batches—most probably owing to the dominance of impurities or decomposition products with much stronger (antiferromagnetic) coupling between the Mn magnetic centers. While we could selectively produce one of the decomposition products, [K(crypt‐222)][Mn(hmds)_3_],^[48]^ and investigate its magnetic susceptibility, as well as for the reactant [Mn(hmds)_2_] (both showing very weak antiferromagnetic couplings), these results did not allow us to draw additional conclusions on the magnetic properties of **2**. So at this stage, we suggest from the calculations that a weak ferromagnetic coupling might be present in the uncommon anion in **2**, but we need to refer to future work for a direct experimental proof of it.

The impact of available d orbitals on the structural features of the anion in **2** became evident by replacing the Mn atoms computationally with Zn that has no open d orbitals. This resulted in a switch from the side‐on to an end‐on bridging Bi_2_^2−^ unit in a Zn−Bi−Bi−Zn zig‐zag conformation (see Figure S8). A calculation of the Zn species starting with a structure according to the one observed in compound **2** does not converge into a local minimum without symmetry restrictions. With symmetry restrictions, a local minimum structure is obtained that is energetically disfavored by 48 kJ mol^−1^ with regard to the end‐on isomer.

**Figure 4:**
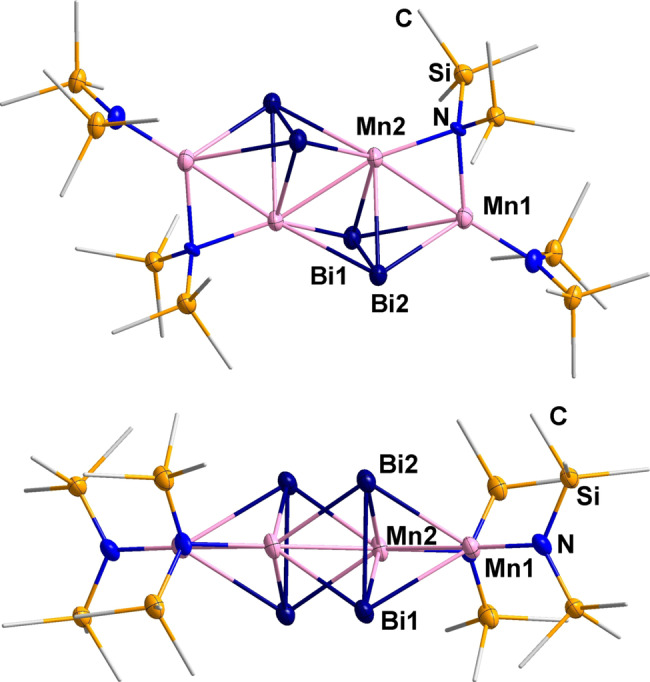
Molecular structure of the anion in **3** in two perpendicular views. Thermal ellipsoids of Bi, Mn, N, and Si atoms are drawn with 50 % probability, with C atoms are drawn as wires. Selected distances in **3** [Å]: Bi−Bi 2.9993(10), Bi−Mn1 2.934(2) and 2.966(2), Bi−Mn2 2.842(2)–2.887(2), Mn1−Mn2 2.720(3), Mn2−Mn2′ 2.804(4), Mn−(μ‐N) 2.108(10) and 2.218(10), Mn−N 2.051(10).

Stirring a suspension of [K(crypt‐222)]_2_(GaBi_3_) ⋅ en or [K(crypt‐222)]_2_(InBi_3_) ⋅ en in THF with [Mn(hmds)_2_] for 30 minutes instead of 5 minutes (as for the formation of **2**) before layering of the filtrate with *n*‐hexane yielded crystals of compound **3**. The molecular structure of the anion in **3** is illustrated in Figure 4 (Supplementary crystallographic figures are provided in the Supporting Information).

Two Bi_2_^2−^ dumbbells act as η^2^‐η^2^‐η^2^‐μ^3^‐bridge to a zig‐zag‐shaped [{Mn(hmds)}_4_]^2+^ unit. Alternatively, the structure of the 8‐atom cluster can be described as two {Bi_2_Mn_3_} *pseudo*‐trigonal bipyramids that are fused by sharing one Mn–Mn edge and being surrounded by four (hmds)^−^ anions. Two of the four (hmds)^−^ ligands act as terminal ligands to the outer Mn atoms (Mn1 and Mn1′), while the two other ligand molecules bridge the Mn1−Mn2 edges opposite to the η^2^‐η^2^‐η^2^‐μ^3^‐Bi_2_^2−^ units. In comparison with the free Bi_2_^2−^ dumbbell, the Bi−Bi distance has again been expanded to 2.9993(10) Å, in agreement with its role as a μ^3^‐bridging ligand (as compared to the μ‐bridging role in the anion in **2**). Hence, this result indicates the most significant involvement of this {Bi_2_} unit in coordination of the transition metal atoms. The anion in **3** can be regarded as a secondary product of the formation of **2**—whose formation might be explained by dimerization of **2** under release of four (hmds)^−^ groups. Further, a 2e^−^ reduction takes place, most probably with the aid of either additional Bi_2_^2−^ or the reactant (TrBi_3_)^2−^ (Tr=Ga, In). Any attempts of monitoring the formation of **2** or **3** was prohibited by the paramagnetic nature of the reaction mixtures as well as immediate decomposition of the compounds under ESI‐MS conditions. Side‐products that were secured during these reactions are [K(crypt)][Mn(hmds)_3_] and Hhmds, and explain the fate of the (hmds)^−^ groups that left the coordination sphere of Mn^2+^ as compared to the original [Mn(hmds)_2_] complex during the formation of **3**. The Hhmds molecules are most likely formed from deprotonation of crystal solvent (en) present in the employed Zintl salts [K(crypt‐222)]_2_‐(TrBi_3_) ⋅ en.

Formally, the transition metal atoms represent a {Mn_4_}^6+^ unit in **3**. The different coordination modes of the (hmds)^−^ groups suggest differences in the formal charges of the individual Mn atoms. The outer Mn atoms are coordinated by a terminal and a μ‐bridging (hmds)^−^ ligand (and one μ^3^‐bridging Bi_2_^2−^ anion), while the two inner Mn atoms are coordinated by one μ‐bridging (hmds)^−^ group (and two μ^3^‐bridging Bi_2_^2−^ units). When counting the effective charge of a terminal (hmds)^−^ anion as −1, that of a μ‐bridging (hmds)^−^ anion as −1/2 , and that of a μ^3^‐bridging Bi_2_^2−^ unit as −2/3 , it is obvious that the outer Mn atoms need to compensate for more negative charges (a total of −21/3 ) than the inner Mn atoms (a total of −11/3 ) in their direct coordination environment (disregarding the additional two negative charges of the entire cluster). This suggests that the outer atoms are closer to a formal +II oxidation state, while the inner ones can be viewed as Mn(+I). The different coordination modes should thus affect the electronic structure and bonding as well as magnetic interactions within these anions. The combination of two Mn^+^ ions (Mn1 and Mn1^i^; *S*=2) with two Mn^2+^ ions (Mn2 and Mn2^i^; *S*=^5^/_2_) can afford a total spin of *S*=0 for a maximum of antiferromagnetic interactions, or a total spin of *S*=9 for ferromagnetic coupling only, or a spin state in between in a more complex case. In theory, this should be distinguishable by magnetic or ESR measurements. However, as the crystals grow on the bottom of the Schlenk tube in very low yields within metal powder, we were not able to isolate enough pure substance for these kinds of experiments so far. DFT calculations were thus undertaken to rationalize the different formal charges and to better understand the electronic situations and potential magnetic coupling schemes in the cluster anion in **3**. For the structure obtained by XRD, we calculated a high‐spin state (hs) with a total of 18 unpaired electrons and a broken‐symmetry (bs) state with alternating surplus of electrons of the spin types (α vs. β) at the four Mn atoms. The bs state turned out to be favored over hs by 75 kJ mol^−1^, which is a strong indication for antiferromagnetic coupling between the magnetic Mn sites in this case. Moreover, the HOMO–LUMO gap is much larger for bs (1.85 eV) than for hs (1.13 eV). Mulliken population analyses indicated that a clear assignment of oxidation states is difficult though. The two inner Mn atoms indeed show a slightly higher population of d orbitals (5.58 electrons each) than the two outer Mn atoms (5.41 electrons each), but a localization procedure yields either five α spin or five β spin orbitals at each of the four Mn atoms. The electrons of the corresponding minority spin are rather localized in bonds to the Bi atoms, which reminds of the situation in **2**. In turn, like in **2** but even more pronounced, the bond order of the Bi−Bi bonds is reduced (Wiberg index 0.87) and the bond length is increased by 1.23 Å as compared to Bi_2_^2−^, in excellent agreement with the experimental observation (Δ*d*=1.78 Å).

Finally, the work indicates the different synthetic behavior of the three binary anions under the given reaction conditions. Attempts to explore this by considering corresponding exchange reactions (see Supporting Information) indicated, however, that the reasons for this observation seem to be manifold. While the different bonding energies within the starting material and in the product molecules may control the course of the formation reactions, the formation of solid by‐products on the one hand and the observation of the products in crystalline form on the other hand seem to be very important parameters that are difficult to model. We refer to future work into this direction, which requires comprehensive considerations of both molecular as well as solid‐state energies and exceeds the scope of this work.

## Conclusion

In the work presented herein, we report a new synthetic approach to Zintl chemistry in common organic solvents, which additionally afforded new multimetallic Zintl clusters. We combined two seemingly contradictory systems—namely salts of binary Tr/Bi Zintl anions (Tr=Ga, In, or Tl), with their known exclusive solubility in highly polar solvents, and the low‐coordinate complex [Mn(hmds)_2_], which is susceptible to such solvents but well‐soluble in THF. We demonstrated that the combination of both in THF not only yields a nice reactive solution instantly, but also leads to the formation of new types of ternary or binary Zintl clusters comprising 6, 4, or 8 metal atoms of the elemental combinations Mn/Tl/Bi or Mn/Bi, respectively. We elucidated in a combined experimental and theoretical study the formation, the geometric, and the uncommon electronic structures of the new clusters [{(hmds)_2_Mn}_2_(TlBi_3_)]^2−^ (in **1**), [{(hmds)_2_Mn}_2_(Bi_2_)]^2−^ (in **2**), and [{(hmds)Mn}_4_(Bi_2_)_2_]^2−^ (in **3**). This—among others—revealed **1** to be the first compound with the (TlBi_3_)^2−^ anion as a ligand to transition metal ions, and indicated the likeliness of weak ferromagnetic interactions of the Mn^2+^ ions via a μ‐Bi_2_^2−^ bridge in the diamond‐shaped bimetallic unit in **2**. Moreover, the anion in **3** represents a very uncommon heteroatomic cluster based on a (formally) mixed‐valent {Mn^I^_2_Mn^II^_2_} unit bridged by two Bi_2_^2−^ units, which together can be viewed as a dimeric version of the anion in **2** upon release of two (hmds)^−^ ligands and a concomitant 2‐e^−^ reduction. This provides an idea of how such binary polyanions grow further. As was shown by ESI mass spectrometry and magnetic measurements the compounds are comparably sensitive—which is in agreement with the observation of relatively low energy barriers calculated for structural changes.

For applying the newly formed clusters in subsequent reactions, future work will also address other types of transition metal ions and ligands involved in the low‐coordinate complexes and the use of other binary p‐block (semi)metal anions. Preliminary studies already indicated that this concept can be successfully expanded to many other d‐block/p‐block elemental combinations.

## Conflict of interest

The authors declare no conflict of interest.

1

## Supporting information

As a service to our authors and readers, this journal provides supporting information supplied by the authors. Such materials are peer reviewed and may be re‐organized for online delivery, but are not copy‐edited or typeset. Technical support issues arising from supporting information (other than missing files) should be addressed to the authors.

Supporting Information

Supporting Information

Supporting Information

## Data Availability

The data that support the findings of this study are available in the supplementary material of this article.
