# Supplementary material for: Reactive Solubilization of Heterometallic Clusters by Treatment of (TrBi3)2− Anions (Tr=Ga, In, Tl) with [Mn{N(SiMe3)2}2]
Source: Angew Chem Int Ed Engl. 2022 Sep 15;61(42):e202210683. doi: 10.1002/anie.202210683 (PMC9825972; doi:10.1002/anie.202210683)
Supplement: Supplementary file 3 — Supporting Information [file ANIE-61-0-s001.pdf]

Angewandte Chemie
Eine Zeitschrift der Gesellschaft Deutscher Chemiker

Supporting Information

Reactive Solubilization of Heterometallic Clusters by Treatment of $(TrBi_3)^{2-}$ Anions (Tr = Ga, In, Tl) with $[Mn\{N(SiMe_3)_2\}_2]$

J. Rienmüller, A. Schmidt, N. J. Yutronkie, R. Clérac, C. G. Werncke, F. Weigend*, S. Dehnen**

Table of Contents

1 Experimental Details 2
2 Single-Crystal Diffraction and Refinement Data 3
3 Micro-X-Ray Fluorescence Spectroscopy (μ-XFS) 6
4 Quantum Chemical Calculations 8
5 Attempts to Measure the Magnetic Behavior of Compound 2 12
6 References 13

1 Experimental Details

1.1 General Methods

All syntheses were performed under exclusion of air and moisture using standard Schlenk or glovebox techniques. Ethane-1,2-diamine (en), was distilled from CaH_2 and stored over 4 Å molecular sieves. Tetrahydrofuran (THF), diethyl ether (Et_2O), *n*-pentane and *n*-hexane, were distilled from potassium and stored over 4 Å molecular sieves. Crypt-222[1] was dried *in vacuo* for 12 hours. Ternary solids $K_5Tr_2Bi_4$ (Tr = Ga, In, Bi) were synthesized and extracted in en as described previously.[2] [Mn(hmds)$_2$] was synthesized by addition of Khmds to $MnCl_2$ in THF according to a literature protocol.[3]

1.2 Preparation of [K(crypt222)]$_2$-[{(hmds)$_2$Mn}$_2$(TlBi$_3$)]·1.5Et$_2$O (1): [K(crypt-222)]$_2$(TlBi$_3$) (30.00 mg, 18.05 µmol) were added to [Mn(hmds)$_2$] (26 mg, 36.09 µmol) in a brown glass vial in a glovebox and dissolved in 2 mL of a 1:1 mixture (v:v) of Et_2O and THF. After stirring for 5 min, the suspension was filtered. The dark brown solution was layered with 2 ml of *n*-hexane and stored at –40°C. After one week, small dark-brown plank-shaped crystals of **1** crystallized (yield: ~10%) at the bottom of the Schlenk tube besides a large amount of colorless crystals of [K(crypt-222)][Mn(hmds)$_3$].

1.3 Preparation of [K(crypt-222)]$_2$[{(hmds)$_2$Mn}$_2$(Bi$_2$)]·4THF (2): [K(crypt-222)]$_2$(GaBi$_3$) (38 mg, 24.87 µmol) or [K(crypt-222)]$_2$(InBi$_3$) (38 mg, 23.27 µmol) were added to [Mn(hmds)$_2$] (35 mg, 48.33 µmol) in a brown glass vial in a glovebox and dissolved in 1.5 mL of THF. After stirring for 5 min, the suspension was filtered. The dark brown solution was layered with 2 ml of *n*-hexane and stored at –40°C. After one week, large dark-brown plank-shaped crystals of **2** (yield: ~30%), which were covered with a powdery precipitate, crystallized at the bottom of the Schlenk tube.

1.4 Preparation of [K(crypt-222)]$_2$[{(hmds)$_2$Mn}$_4$(Bi$_2$)$_2$] (3): [K(crypt-222)]$_2$(GaBi$_3$) (38 mg, 24.87 µmol) or [K(crypt-222)]$_2$(InBi$_3$) (38 mg, 23.27 µmol) were added to [Mn(hmds)$_2$] (40.00 mg, 55.23 µmol) in a brown glass vial in a glovebox and dissolved in 1.5 mL of THF. After 30 min, the suspension was filtered. The dark brown solution was layered with 2 ml of *n*-hexane and stored at –40°C. After a few days, small dark-red irregularly shaped crystals of **3** (yield: ~15%), which were covered with a powdery precipitate, appeared at the bottom of the Schlenk tube.

2 Single-Crystal Diffraction and Refinement Data

2.1 Crystal Measurement and Refinement Details

The data sets were collected on a Bruker D8 Quest with microfocus source emitting $Mo_{K\alpha}$ radiation (λ = 0.71073Å) and a Photon 100 detector at T = 100 K. The structures were solved by dual space methods of SHELXT-2018/2 within the Olex2-1.3 software[4–6] and refined using least-squares procedures on a F^2 with SHELXL-2018/3 in Olex2.[6,7,4] General crystallographic data are listed in Table S1.

Table S1: Crystal data and details of the structure determination of 1 – 3.

Compound	1·1.5Et₂O	2·4THF	3
empirical formula	$C_{64.50}H_{146.50}Bi_3K_2Mn_2N_8O_{12.50}Si_8Tl$	$C_{68}H_{160}Bi_2K_2Mn_2N_8O_{14}Si_8$	$C_{60}H_{144}Bi_4K_2Mn_4N_8O_{12}Si_8$
chemical formula	$[K(crypt222)]_2[\{(hmds)_2Mn\}_2(TlBi_3)]\cdot 1.5Et_2O$	$[K(crypt\text{-}222)]_2[\{(hmds)_2Mn\}_2(Bi_2)]\cdot 4THF$	$[K(crypt\text{-}222)]_2[\{(hmds)Mn\}_4(Bi_2)_2]$
emp. formula weight [g mol^{-1}]	2488.05	2144.79	2528.42
temperature [K]	100	100	100
crystal color, shape	dark-red irregular block	dark brown plank	dark black irregular block
crystal system	Triclinic	monoclinic	triclinic
space group	$P\bar{1}$ (Nr. 2)	$C2/c$ (Nr.15)	$P\bar{1}$ (Nr. 2)
a [Å]	14.532(2)	26.3859(10)	11.8800(17)
b [Å]	15.288(3)	16.6339(5)	14.941(3)
c [Å]	26.852(4)	26.1946(10)	16.334(5)
α [°]	82.277(6)	90	66.01(3)
β [°]	74.809(4)	112.4840(10)	73.461(14)
γ [°]	62.273(5)	90	66.837(9)
V [Å^3]	5095.5(14)	10622.9(7)	2408.5(10)
Z	2	4	1
ρ_{calc} [g cm^{-3}]	1.622	1.341	1.743
μ [mm^{-1}]	7.204	3.755	8.021
F(000)	2456	4408	1238
crystal size [mm^3]	0.412 × 0.278 × 0.1	0.088 × 0.141 × 0.385	0.122 × 0.133 × 0.25
radiation	$Mo_{K\alpha}$ (λ = 0.71073 Å)	$Mo_{K\alpha}$ (λ = 0.71073 Å)	$Mo_{K\alpha}$ (λ = 0.71073 Å)
2θ range [°]	4.32 – 55.98	4.90 – 52.16	3.91 – 51.00
Index ranges	$-18 \le h \le 19$, $-20 \le k \le 20$, $-35 \le l \le 35$	$-32 \le h \le 32$, $-20 \le k \le 20$, $-32 \le l \le 32$	$-14 \le h \le 14$, $-18 \le k \le 18$, $-19 \le l \le 19$
absorption correction type	multi-scan	multi-scan	multi-scan
reflections collected	156719	90054	33278
ind. reflections / R_{int} / R_{sigma}	24433 / 0.0583 / 0.0425	10509 / 0.0446 / 0.0229	8633 / 0.0646 / 0.0642
restraints / parameters	269 / 1069	6 / 492	48 / 454
Final R indexes [$I \ge 2\sigma(I)$]	R_1= 0.0332, wR_2= 0.0707	R_1= 0.0200, wR_2= 0.0407	R_1= 0.0611, wR_2= 0.153
Final R indexes [all data]	R_1 = 0.0550, wR_2 = 0.0794	R_1 = 0.0271, wR_2 = 0.0425	R_1 = 0.1113, wR_2 = 0.179
goodness–of–fit on F^2	1.013	1.042	1.148
max peak / hole [e Å^{-3}]	1.556 / –1.734	0.452 / – 0.541	4.609 / –2.377
CCDC number	2190219	2190220	2190221

All non-hydrogen atoms were refined using anisotropic displacement parameters. All hydrogen atoms were refined by using a riding model. Absorption correcting was carried out using MULTISCAN. Supplementary structural figures are shown in Figures S1-S4. The structures were drawn with DIAMOND.[8] They are shown with displacement ellipsoids at the 50% probability level for non-hydrogen atoms. Hydrogen atoms are not shown for clarity.

2.2 Supplementary Structural Figures

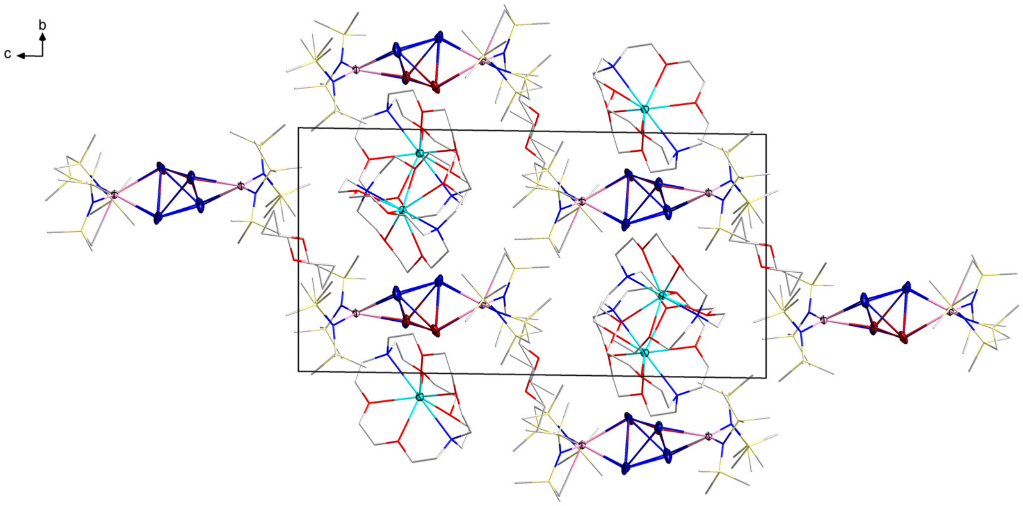

Figure S1: Unit cell of compound 1·1.5Et$_2$O. Displacement ellipsoids are shown with 50% probability for non-hydrogen atoms; hydrogen atoms are not shown for clarity.

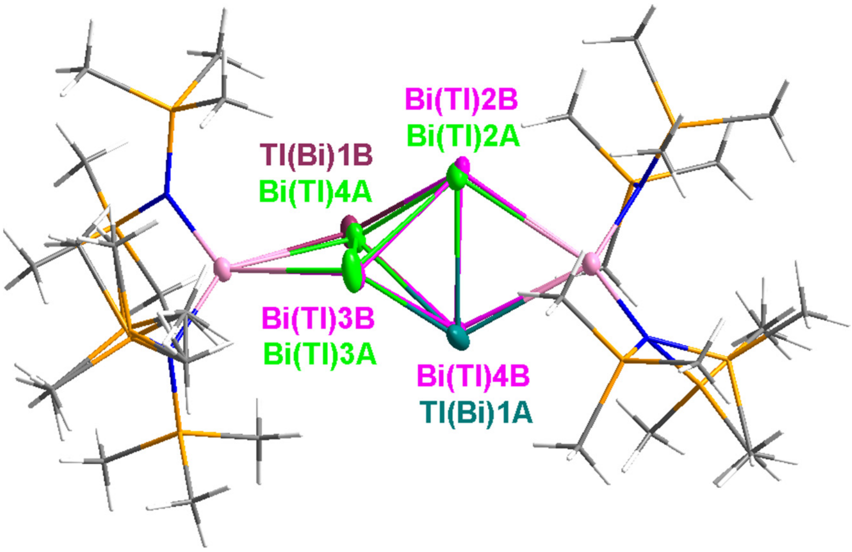

Figure S2: Molecular structure of the anion in compound 1·1.5Et$_2$O, outlining the disorder of the {TlBi$_3$} unit into a set of A split positions (green/teal) and a set of B split position (pink/plum). Displacement ellipsoids of metal atoms are shown with 50% probability.

Refinement of **1**·1.5Et$_2$O led to best results, when considering a statistical disorder of the *pseudo*-tetrahedral unit over two very slightly differing sets of atomic sites (A and B) with corresponding split positions. As Tl and Bi atoms cannot be distinguished by standard X-ray

diffraction experiments, all involved atomic sites were assigned a 0.125 occupancy by Tl atoms and a 0.375 occupancy by Bi atoms. For both sets, A and B, one of the atoms was randomly picked to represent the Tl atom in Figure S2. The disorder cannot be reasonably modeled by considering a larger unit cell or a lower symmetry space group; structure solution and refinement in $P1$ led to the same finding.

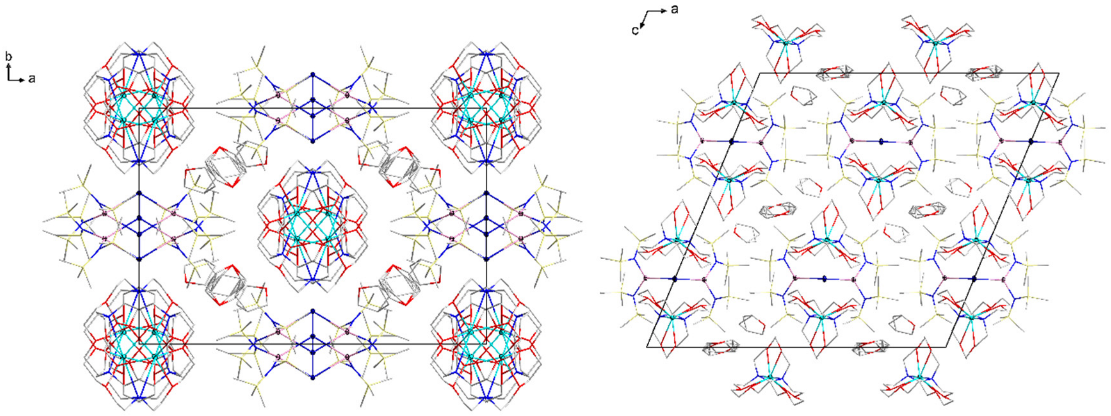

Figure S3: Fragment of the crystal structure of compound 2·4THF viewed along the crystallographic *c* and *b* axes (left-hand side and right-hand side, respectively). Displacement ellipsoids are shown with 50% probability for non-hydrogen atoms; hydrogen atoms are not shown for clarity.

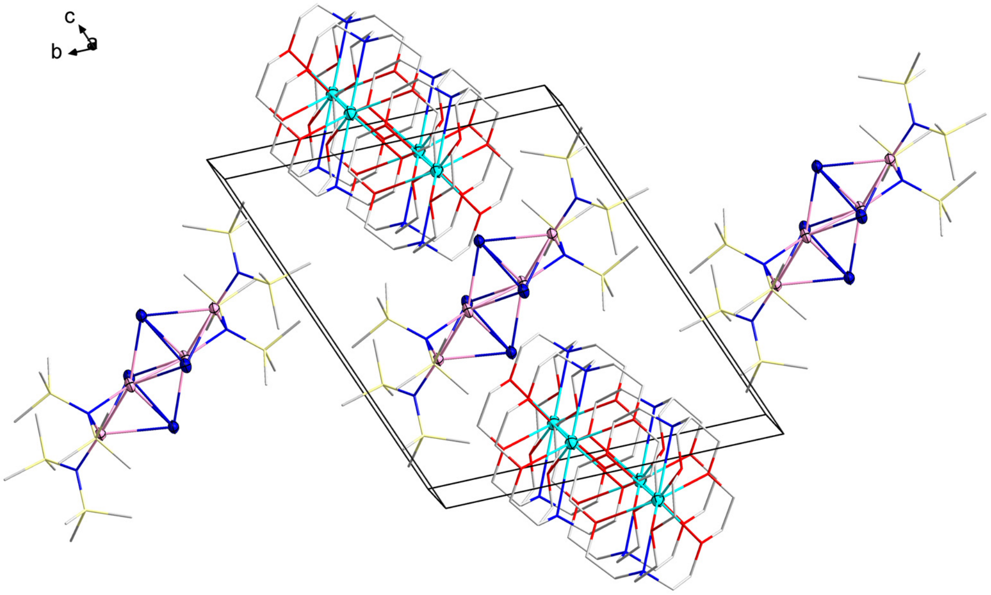

Figure S4: Fragment of the crystal structure of compound 3. Displacement ellipsoids are shown with 50% probability for non-hydrogen atoms; hydrogen atoms are not shown for clarity.

3 Micro-X-Ray Fluorescence Spectroscopy (μ-XFS)

All μ-XFS measurements were performed on a Bruker M4 Tornado, equipped with an Rh-target X-ray tube and a silicon drift detector. Quantification of the elements is achieved through deconvolution of the spectra. The results are summarized in Table S2. The rhodium content from the target is omitted from the quantification results. Figures S5-S7 show the spectra for single crystals of compounds **1**·1.5Et_2O, **2**·4THF, and **3**, respectively, along with the results of the deconvolution algorithm. Silicon and potassium contents are notoriously difficult to detect in correct amounts by means of μ-XFS, for which the values are not given here.

Table S2: Summary of the Micro X-Ray Fluorescence Spectroscopy Results

Compound	Element	Series	Element wt%	Atom %	Element ratio	
					Exp.	Calc.
**1**·1.5Et_2O	Mn	K	10.67	22.58	2	2.03
	Tl	L	19.53	11.11	1	1
	Bi	L	58.86	32.73	3	2.94
**2**·4THF	Mn	K	19,15	28,68	1	1.16
	Bi	L	60,68	23.88	1	1
**3**	Mn	K	16.84	30.07	1	1
	Bi	L	68.00	31.91	1	1.06

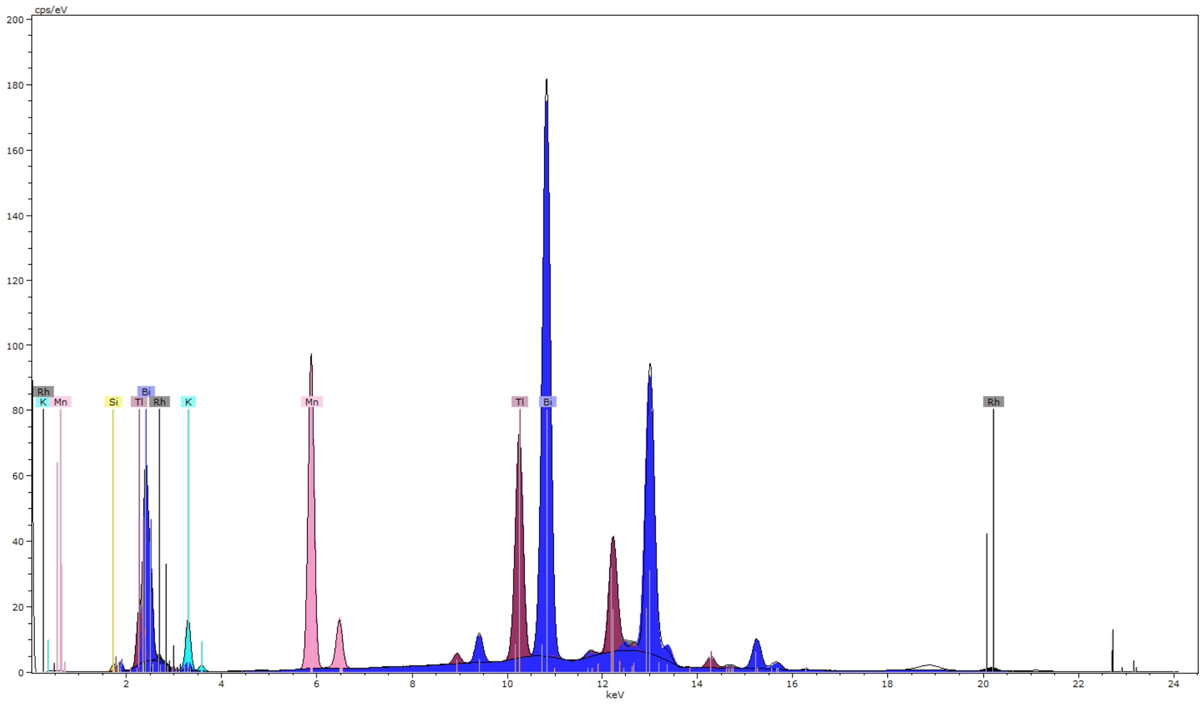

Figure S5: Micro X-ray fluorescence spectrum of compound 1·1.5Et_2O with the results of the deconvolution algorithm. The color code refers to the one used in the crystal structure figures: K (sky blue), Bi (blue), Mn (rose), Tl (red-brown), Si (yellow).

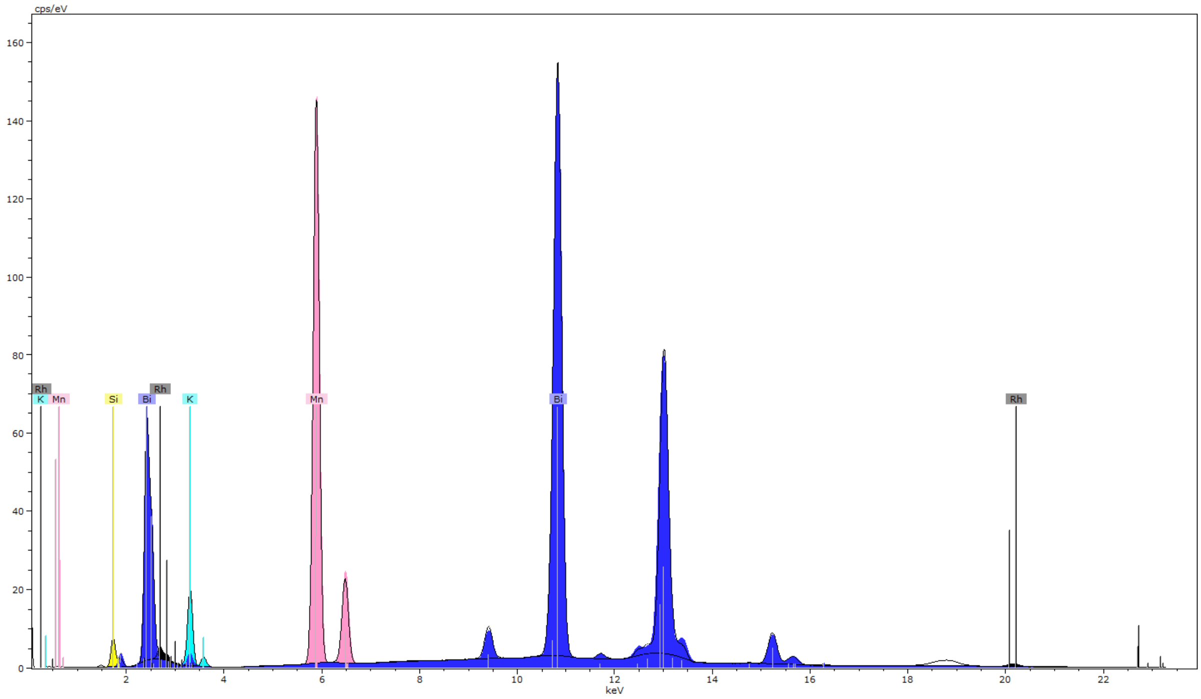

Figure S6: Micro X-ray fluorescence spectrum of compound 2·4THF with the results of the deconvolution algorithm. The color code refers to the one used in the crystal structure figures: K (sky blue), Bi (blue), Mn (rose), Si (yellow).

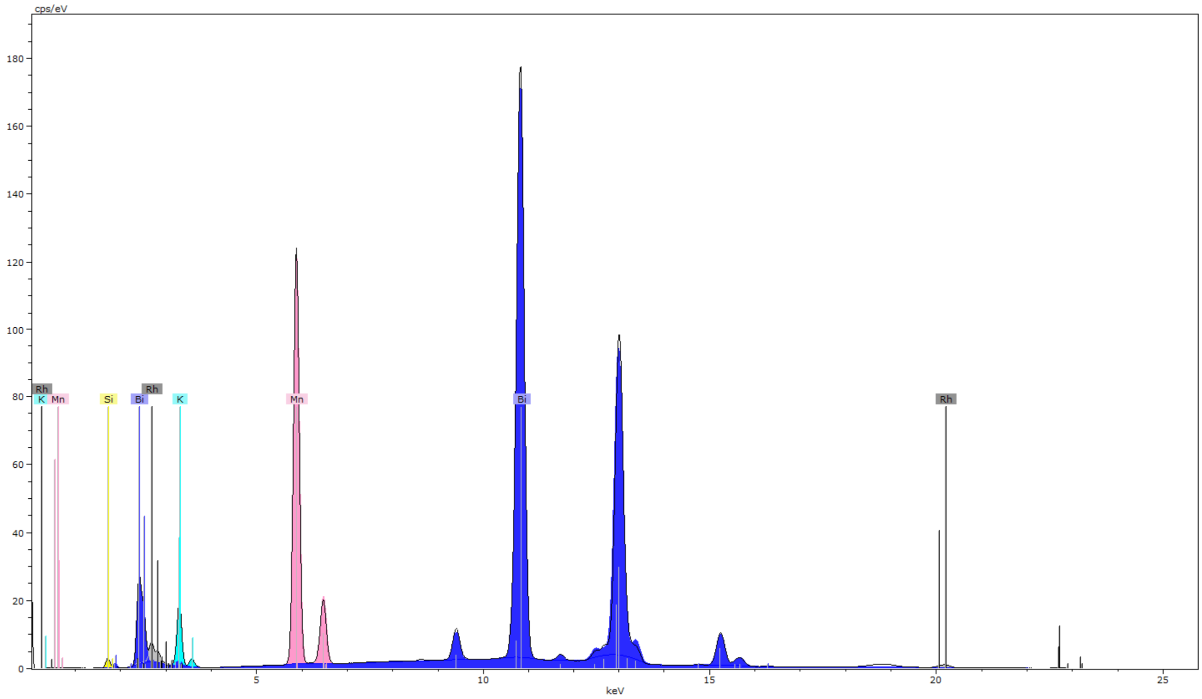

Figure S7: Micro X-ray fluorescence spectrum of compound 3 with the results of the deconvolution algorithm. The color code refers to the one used in the crystal structure figures: K (sky blue), Bi (blue), Mn (rose), Si (yellow).

4 Quantum Chemical Calculations

4.1 Methods

Density functional calculations have been performed using the program Turbomole (Version 7.5.) with the functional TPSSh.[9] The basis set def2-TZVP[10] has been used with the corresponding auxiliary bases and effective core potentials (ECP-60) for thallium and bismuth as well as an ECP-28 for indium.[11] Counter ions have been modeled with the conductor-like screening model (COSMO)[12] using standard settings and an infinite dielectric constant. Force constants and vibrational spectra were calculated using the aoforce program. The absence of any imaginary frequencies proves the structure to be a local minimum. Population analyses were performed using the method of Mulliken,[14] bond orders were calculated using the method of Mayer and Wiberg,[15] and molecular orbitals were plotted with Chemcraft (Version 1.8).[16]

4.2. Structural Data, Mulliken Charges, and Numbers of Unpaired Electrons (Mn)

Table S3: Comparison of experimental and calculated interatomic distances (in Å) of the cluster anion in 1. Atom numbers refer to the ones used in Figure 2 in the main document.

Bond	Experimental	Calculated
Bi–Bi		2.974 – 3.032
Bi–Tl		3.114 – 3.283
Bi/Tl–Bi/Tl	2.805(3) – 3.314(3)	
Mn–Bi		3.019 – 3.090
Mn–Tl		3.218
Mn–Bi/Tl	2.966(3) – 3.105(3)	
Mn–N	2.037(4) – 2.051(3)	2.034 – 2.063

Table S4: Comparison of experimental and calculated interatomic distances (in Å) of the cluster anion in 2. Atom numbers refer to the ones used in Figure 2 in the main document.

Bond	Experimental	Calculated
Bi1–Bi2	2.9022(2)	2.874
Bi1–Mn1	2.9617(3)	2.928
Bi2–Mn1	2.9712(3)	2.928
Mn1–N2	2.0659(19)	2.056
Mn1–N1	2.0692(18)	2.056

Table S5: Comparison of experimental and calculated interatomic distances (in Å) of the cluster anion in 3. Atom numbers refer to the ones used in Figure 4 in the main document.

Bond	Experimental	Calculated
Bi1–Bi2	2.9993(10)	2.969
Bi1–Mn2	2.887(2)	2.864
Bi1–Mn2′	2.842(2)	2.832
Bi1–Mn1	2.966(2)	2.973
Bi2–Mn2	2.877(2)	2.856
Bi2–Mn2′	2.8468(19)	2.846
Bi2–Mn1	2.934(2)	2.953
Mn2–Mn2′	2.804(4)	2.807
Mn1–Mn2	2.720(3)	2.724

Table S6: Mulliken charges at the bismuth atoms before and after the coordination of $(TrBi_3)^{2-}$ (Tr = Ga, In, Tl) to $Mn(hmds)_2$ and number of unpaired electrons (nue) at manganese in $L_2(TrBi_3)^{2-}$ (high-spin configuration; L = $\{(hmds)_2Mn\}$).

	Mulliken charge (Bi)			nue (Mn)	
$(TlBi_3)^{2-}$	–0.57			–	
$[L_2(TlBi_3)]^{2-}$	–0.36	–0.38	–0.39	4.87	4.92
$(InBi_3)^{2-}$	–0.53			–	
$[L_2(InBi_3)]^{2-}$	–0.33	–0.33	–0.33	4.87	4.92
$(GaBi_3)^{2-}$	–0.48			–	
$[L_2(GaBi_3)]^{2-}$	–0.27	–0.28	–0.30	4.88	4.89

Table S7: Mulliken charges at the bismuth atoms before and after the coordination of $(Bi_2)^{2-}$ anions to $Mn(hmds)_2$ and number of unpaired electrons (nue) at manganese in $[L_2(Bi_2)\}^{2-}$ (high-spin configuration; L = $\{(hmds)_2Mn\}$).

	Mulliken charge (Bi)		nue (Mn)	
$(Bi_2)^{2-}$	−1		–	
$[L_2(Bi_2)]^{2-}$	−0.58	−0.58	4.69	4.69

Table S8: Mulliken charges at the bismuth atoms before and after the coordination of two $(Bi_2)^{2-}$ anions to one $\{Mn(hmds)_2\}$ and one $\{Mn(hmds)_2\}^+$ moiety and number of unpaired electrons (nue) at the manganese atoms in $[L_2L'_2(Bi_2)_2\}^{2-}$ (L = $\{(hmds)_2Mn\}$; L′ = $\{(hmds)Mn\}^+$). Positive values indicate alpha spins, negative values indicate beta spins.

	Mulliken charge (Bi)		nue (Mn)			
$(Bi_2)^{2-}$	−1		–			
$[L_2(Bi_2)]^{2-}$	−0.42	−0.40	4.79	−4.38	4.38	−4.79

4.2 Calculated Structure of a Hypothetical Zn Analogue of Compound 2

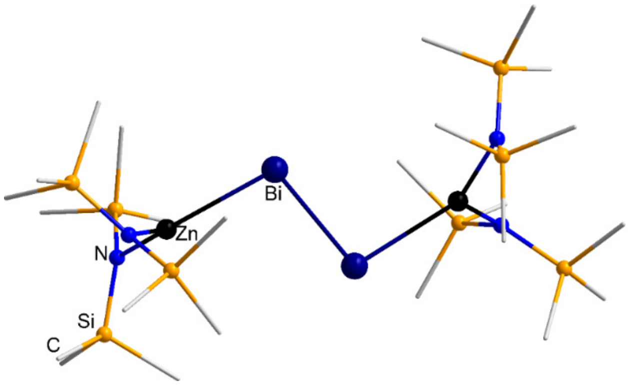

Figure S8: Calculated end-on coordination of Bi_2^{2-} upon interaction with $[Zn(hmds)_2]$ units.

4.1 Considerations on the Different Reaction Behavior of $(GaBi_3)^{2-}$, $(InBi_3)^{2-}$, and $(TlBi_3)^{2-}$

We used quantum chemical calculations to explore possible reasons why the three binary anions behave so differently in the syntheses explored in this work.

As an attempt to explore why **1** is observed for Tr = Tl, while starting materials with Tr = In or Ga afford the anion in **2**, we calculated the energies for a reaction affording the anion in **2** from that in **1** (and their hypothetical homologues), as shown in equation (1):

$$[\{(hmds)_2Mn\}_2(TrBi_3)]^{2-} \rightarrow [\{(hmds)_2Mn\}_2(Bi_2)]^{2-} + Tr + Bi \qquad (1)$$

Corresponding reaction energies are 461, 483, or 525 kJ/mol for Tr = Tl, In, or Ga, respectively. The reactions thus show a strong preference for the left-hand side (hence, the anion in **1**, and its homologues) in particular for Ga, which disagrees with the experimental observation. Matters change however, when accounting for the experimental observation of metallic deposit by including the cohesive energy of 182, 243, or 277 kJ/mol (Tr = Tl, In, or Ga), and 207 kJ/mol (Bi) at the right-hand side. The left-hand side now is only slightly preferred by 72, 33, or 41 kJ/mol (Tr = Tl, In, or Ga), with the In and Ga cases being less endoenergetic, and thus the fragmentation is more likely to occur. Additionally, one needs to take into consideration the driving force of crystallization as $[K(crypt\text{-}222)]^+$ salts; it is very likely that the (negative) lattice energies associated with the crystal formation easily overcompensate the small (positive) numbers for the calculated reaction energies. In summary these studies mainly demonstrate the subtle differences between the compounds of the three triel elements, and also indicate the necessity to account for solid precipitates or crystallization in order to explain the experimental findings.

The formation of the anion in **2** was also studied in comparison with the other elemental combinations according to equation (2):

$$(TrBi_3)^{2-} + 2\ [Mn(hmds)_2] \rightarrow [\{(hmds)_2Mn\}_2(Bi_2)]^{2-} + Tr + Bi \qquad (2)$$

Under consideration of the cohesive energies of Tr, as done for equation (1), reaction energies are +2.5, –34.7, or –35.5 kJ/mol (Tr = Ga, In, Tl). Thus, the preference of this structure for Ga and In relative to Tl is obvious and in agreement with the experimental observations – even without consideration of lattice energies in this case, which are likely to enhance the trend for the reasons given above (and the assumption of a relatively stable salt of the highly symmetric $(TrBi_3)^{2-}$ anion).

5 Attempts to Measure the Magnetic Behavior of Compound 2

The magnetization measurements were performed on a Quantum Design MPMS-XL SQUID magnetometer operating between 1.8 and 400 K and applied dc fields of up to 7 T. The measurements were performed on microcrystalline samples (2.1, 12.9, 10.6, 9.9 and 16.2 mg) sealed in double polypropylene/polyethylene bags (with a typical size of 3 × 0.5 × 0.02 cm^3, and mass of 32.5, 18.4, 24.9, 34.3, 27.4 mg respectively) under argon. The data were corrected for the intrinsic diamagnetic contributions of the sample and the sample holder.

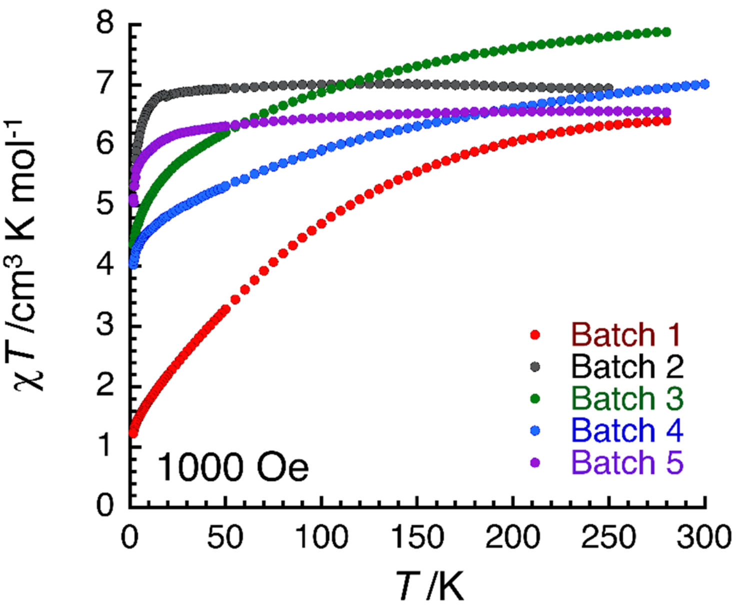

Figure S9: Temperature dependence of the χT product for the five different batches of the product obtained at the formation of compound 2 (see discussion in the main text) at 0.1 T (χ is defined as M/H per mole of complex).

6 References

[1] 4,7,13,16,21,24-Hexaoxa-1,10-diazabicyclo[8.8.8]hexacosane.

[2] a) L. Xu, S. C. Sevov, *Inorg. Chem.* **2000**, *39*, 5383; b) N. Lichtenberger, Y. J. Franzke, W. Massa, F. Weigend, S. Dehnen, *Chem. Eur. J.* **2018**, *24*, 12022; c) N. Lichtenberger, N. Spang, A. Eichhöfer, S. Dehnen, *Angew. Chem.* **2017**, *129*, 13436.

[3] H. Bürger, U. Wannagat, *Monathefte für Chemie* **1964**, *95*, 1099.

[4] O. V. Dolomanov, L. J. Bourhis, R. J. Gildea, J. A. K. Howard, H. Puschmann, *J. Appl. Crystallogr.* **2009**, *42*, 339.

[5] G. M. Sheldrick, *Acta Crystallogr., Sect. A: Found. Adv.* **2015**, *71*, 3.

[6] G. M. Sheldrick, *Acta Crystallogr., Sect. A: Found. Adv.* **2008**, *64*, 112.

[7] a) G. M. Sheldrick, *Acta Crystallogr., Sect. C: Struct. Chem.* **2015**, *71*, 3; b) G. M. Sheldrick, *ShelXL-2018*, Universität Göttingen, Göttingen (Germany), **2018**.

[8] K. Brandenburg, *Diamond*, Crystal Impact GbR, Bonn, Germany, **2021**.

[9] V. N. Staroverov, G. E. Scuseria, J. Tao, J. P. Perdew, *J. Chem. Phys.* **2003**, *119*, 12129.

[10] F. Weigend, *Phys. Chem. Chem. Phys.* **2006**, *8*, 1057.

[11] B. Metz, H. Stoll, M. Dolg, *J. Chem. Phys.* **2000**, *113*, 2563.

[12] a) A. Klamt, G. Schüürmann, *J. Chem. Soc., Perkin Trans. 2* **1993**, 799. b) A. Schäfer, A. Klamt, D. Sattel, J. C. W. Lohrenz, F. Eckert, *Phys. Chem. Chem. Phys.* **2000**, *2*, 2187.

[13] R. S. Mulliken, *J. Chem. Phys.* **1955**, *23*, 2338.

[14] I. Mayer, Chem. Phys. Lett. **1983**, *97*, 270.

[15] Chemcraft - graphical software for visualization of quantum chemistry computations. https://www.chemcraftprog.com.
